# Facile Assembly of Thermosensitive Liposomes for Active Targeting Imaging and Synergetic Chemo-/Magnetic Hyperthermia Therapy

**DOI:** 10.3389/fbioe.2021.691091

**Published:** 2021-08-04

**Authors:** Yanli An, Rui Yang, Xihui Wang, Yong Han, Gang Jia, Chunmei Hu, Zhiyuan Zhang, Dongfang Liu, Qiusha Tang

**Affiliations:** ^1^Jiangsu Key Laboratory of Molecular and Functional Imaging, Department of Radiology, Zhongda Hospital, Medical School of Southeast University, Nanjing, China; ^2^Research Institute for Reproductive Health and Genetic Diseases, The Affiliated Wuxi Maternity and Child Health Care Hospital of Nanjing Medical University, Wuxi, China; ^3^School of Medicine, Southeast University, Nanjing, China; ^4^Department of Tuberculosis, The Second Affiliated Hospital of Southeast University (The Second Hospital of Nanjing), Nanjing, China; ^5^Department of Neurosurgery, Nanjing Jinling Hospital, School of Medicine, Nanjing University, Nanjing, China

**Keywords:** CD90, combined therapy, imaging, LCSCs, hyperthermia therapy

## Abstract

Cancer stem cells (CSCs) are thought to be responsible for the recurrence of liver cancer, highlighting the urgent need for the development of effective treatment regimens. In this study, 17-allylamino-17-demethoxygeldanamycin (17-AAG) and thermosensitive magnetoliposomes (TMs) conjugated to anti-CD90 (CD90@17-AAG/TMs) were developed for temperature-responsive CD90-targeted synergetic chemo-/magnetic hyperthermia therapy and simultaneous imaging *in vivo.* The targeting ability of CD90@DiR/TMs was studied with near-infrared (NIR) resonance imaging and magnetic resonance imaging (MRI), and the antitumor effect of CD90@17-AAG/TM-mediated magnetic thermotherapy was evaluated *in vivo*. After treatment, the tumors were analyzed with Western blotting, hematoxylin and eosin staining, and immunohistochemical (IHC) staining. The relative intensity of fluorescence was approximately twofold higher in the targeted group than in the non-targeted group, while the *T*_2_ relaxation time was significantly lower in the targeted group than in the non-targeted group. The combined treatment of chemotherapy, thermotherapy, and targeting therapy exhibited the most significant antitumor effect as compared to any of the treatments alone. The anti-CD90 monoclonal antibody (mAb)-targeted delivery system, CD90@17-AAG/TMs, exhibited powerful targeting and antitumor efficacies against CD90^+^ liver cancer stem cells *in vivo*.

## Introduction

Tumorigenesis and heterogeneity, progression, and recurrence of tumors are largely caused by a small subpopulation of tumor cells with stem cell properties, known as the cancer stem cells (CSCs) (Chen et al., 2013). The conventional anticancer drugs generally affect normal cancer cells; however, recurrence is highly frequent owing to the activity of residual CSCs that are resistant to radiotherapy and chemotherapy. Some of the resistance mechanisms involve the dormant or slow-growing phase of the cell cycle such as those that target the ATP-binding cassette (ABC) superfamily (Schätzlein, 2006; Kobayashi et al., 2014; Moitra, 2015). Thus, therapeutic strategies targeting the minor population of CSCs may be one of the most promising approaches for the treatment of resistant tumors.

In general, drugs effective against CSCs may face difficulty in entering and accumulating in the tumor where CSCs are located, resulting in inefficient therapeutic outcomes. Nanoparticles conjugated with specific CSC biomarkers have been recently successfully synthesized to selectively eliminate CSCs (Wang et al., 2013). Wang et al. (2012) showed that the delivery of suicide gene and doxorubicin through CD44-conjugated liposomal nanoparticles that specifically target CD44^+^ tumorigenic cells of hepatocellular carcinoma (HCC) resulted in the induction of apoptosis and inhibition of tumor growth. Furthermore, Rao et al. (2015) developed a doxorubicin-encapsulated polymeric nanoparticle covered with chitosan to target and kill CD44^+^ mammary cancer stem-like cells. This drug delivery system was found to be effective in eliminating cancer stem-like cells, decreasing the tumor volume, and exhibiting low systemic toxicity. Hence, the development of nanoparticles specifically targeted to affect CSC function and maintenance may be an attractive option to cure cancer such that these particles directly target and kill CSCs, reduce systemic toxicity, allow better tolerance to a larger drug dose and longer course, and eliminate the risk of metastasis and tumor recurrence.

At present, therapeutic strategies for CSC-targeted therapy overlap with the multidisciplinary therapies employed for the elimination of non-CSCs. These cells represent a considerable population of tumor cells and play a significant role in CSC growth regulation (Ye et al., 2014). Therefore, the eradication of differentiated cells in tumors may provide a synergetic effect for the clearance of CSCs. CD90 is an important marker for liver cancer stem cells (LCSCs), and study has shown that the expression of CD90 was related to the formation, growth, metastasis, and drug resistance of liver cancer. This makes the treatment of targeting CD90^+^ LCSCs of important practical significance (Liu et al., 2020). It is important and of potential clinical value to find novel therapeutic approaches that specifically kill CSCs and serve as effective supplementary treatments to reduce tumor recurrence and metastasis by improving the therapeutic response. Magnetic hyperthermia is the delivery of magnetic nanoparticles into the target tissue heated to 41–46°C with an alternating current magnetic field (AMF), an adjuvant strategy in cancer treatment (Grumezescu et al., 2014). Thermotherapy has a strong anticancer activity when combined with other treatments (Paunesku et al., 2015; Wang et al., 2019). Thus, magnetic thermotherapy is a novel technique to heat the deep-seated tumors; however, its application is limited owing to its inability to target tumors (Rao et al., 2016). Thermosensitive magnetoliposomes (TMs) are promising agents in the field of targeted liposomes, as these particles render the treatment more effective and safer based on the dual effect of liposomes and magnetic thermotherapy (Wang et al., 2018). In addition, drug-loaded TMs surface modified with a targeting ligand may enhance the accumulation and release of the drug at the target site upon exposure to AMF, serving as a novel approach for targeted liposomal therapeutics (Deshpande et al., 2013).

In our previous study, we prepared anti-CD90 monoclonal antibody (mAb)-targeted TMs encapsulating heat shock protein (HSP) inhibitor, 17-allylamino-17-demethoxygeldanamycin (17-AAG) (CD90@17-AAG/TMs), to target and kill CD90^+^ LCSCs *in vitro* (Yang et al., 2015). In combination with magnetic thermotherapy, CD90@17-AAG/TMs showed good targeting efficiency against CD90^+^ LCSCs and effectively killed these cells *in vitro*. In addition, the upregulation of HSP90 by heat treatment caused thermoresistance *in vitro* and exerted a direct impact on the effectiveness of hyperthermia therapy. HSP inhibitor 17-AAG may reverse thermoresistance and promote apoptosis of CD90^+^ LCSCs *in vitro* (Yang et al., 2015). We collectively studied the targeting property of CD90@17-AAG/TMs in HCC-bearing mice and explored the antitumor effect and mechanism of CD90@17-AAG/TMs against CD90^+^ LCSCs under hyperthermic conditions *in vivo*.

## Materials and Methods

### Cells and Animals

The HCC cell line Huh7 was purchased from the Institute of Biochemistry and Cell Biology (Shanghai, China). CD90^+^ LCSCs were sorted from Huh7, as previously reported (Yang et al., 2015). BALB/c nude mice (female, average weight: 20 g, 5 weeks old) were purchased from the Comparative Medicine Centre of Yangzhou University (Yangzhou, Jiangsu, China).

### Synthesis of CD90@17-AAG/TMs

We prepared 17-AAG/TMs using the rotary evaporator hydration method, as previously reported (Yang et al., 2015). 17-AAG-loaded thermosensitive liposomes (17-AAG/TSLs) comprised dipalmitoylphosphatidylcholine (DPPC), 17-AAG, cholesterol, and 1,2-distearoyl-sn-glycero-3-phosphoethanolamine-N-(methoxy[polyethylene glycol]-2000) n, (PEG2000-DSPE) (54:6:1:1 m/m). CD90@17-AAG/TMs were synthesized as described in our previous report (Deshpande et al., 2013). Anti-CD90 mAb-targeted TMs encapsulating DiR (CD90@DiR/TMs) were prepared using a similar method. The average size of the liposomes used in this study was approximately 130 nm. CD90@17-AAG/TMs were stable in phosphate-buffered saline when stored at 4°C. The phase transition temperature of CD90@17-AAG/TMs was 41.9°C. The liposomes showed good temperature-sensitive release properties at temperatures higher than the phase transition temperature. CD90@17-AAG/TMs could reach the effective thermotherapy temperature of 41°C–44°C upon exposure to AMF.

### Establishment of Tumor-Bearing Mice Model With CD90^+^ LCSCs

Tumors were developed in BALB/c nude mice with a subcutaneous injection of 1 × 10^5^ CD90^+^ LCSCs into the hindlimb. The experimental analysis was initiated once the tumor volume reached 200 mm^3^. Mice were housed in the Sterile Barrier System of the Medical School, Southeast University, Nanjing, China.

### Targeting Ability of CD90@TMs Against CD90^+^ LCSCs *in vivo*

#### Near-Infrared Imaging

Irrelevant mAb-modified DiR/TMs (IgG@DiR/TMs) were used as the control for the targeting experiment. The mice were randomly divided into three groups (five mice per group) as follows: IgG@DiR/TMs, DiR/TMs, and CD90@DiR/TMs. Mice were intravenously injected with DiR at 0.2 mg/kg. Near-infrared (NIR) images were acquired with the Maestro *in vivo* optical imaging system (excitation: 748 nm, emission: 780 nm, exposure time: 400 ms; Caliper Life Sciences, Hopkinton, MA, United States) at preinjection and at 1, 4, 6, 8, 24, 48, and 72 h post-injection and analyzed using the Maestro 2.10.0 software (Caliper Life Sciences, Hopkinton, MA, United States). The relative fluorescence intensity of the tumor was calculated as follows:

Relative fluorescence intensity = fluorescence intensity of the tumor/fluorescence intensity of the muscle.

The fluorescence intensity was measured by manually drawing a region of interest (ROI) within the tumor and muscle areas.

#### Magnetic Resonance Imaging

Based on the treatment, mice were divided into three groups (five mice per group) as follows: IgG@DiR/TMs, DiR/TMs, and CD90@DiR/TMs. The agents were administered through the tail vein under anesthesia (10 mg Fe/kg). The magnetic resonance imaging (MRI) of the tumor was performed at 1, 6, 8, 24, 48, and 72 h post-injection. The mapping sequence for the tumor was as follows: multi-slice multi-echo *T*_2_-weighted imaging (MSME-T2WI): field of view (FOV) = 35 mm × 35 mm, repetition time (TR) = 3000 ms, echo time (TE) = 20 ms, slice thickness = 0.8 mm, and matrix = 256 × 256; FLASH-T2^∗^ sequence: FOV = 35 mm × 35 mm, TR = 408 ms, TE = 3.5 ms, slice thickness = 0.8 mm, and matrix = 256 × 256. The relative signal intensity of the tumor was calculated as follows:

Relative signal intensity = *T*_2_ value of the tumor/*T*_2_ value of the muscle

where *T*_2_ = relaxation times measured by manually drawing an ROI within the tumor and muscle areas.

#### Prussian Blue Staining

After the imaging assays, mice were euthanized. Tumor tissues were excised and fixed in 10% neutral-buffered formalin. Tissues were embedded in paraffin, and 4-μm thick sections were obtained. The sections were successively stained by Prussian blue for ferric ions and nuclear fast red for the cell nucleus and examined using an optical microscopy.

### *In vivo* Targeted Therapy of CD90@17-AAG/TMs

Once the tumor volume reached approximately 200 mm^3^, CD90^+^ LCSC-bearing mice were randomly divided into eight groups (12 mice per cohort) as shown in Table 1. After receiving anesthesia, mice were injected with different liposomes (10 mg Fe/kg, 40 mg 17-AAG/kg) in the caudal vein, as previously reported (Xie et al., 2014, 2016). The frequency of the injection was once a day. After 24 h, hyperthermia groups were placed on AMF (*f* = 200 kHz; *I* = 20 A) for 60 min every other day. Seven days later, half of the mice in each group were euthanized and weighed. The tumors were sectioned, followed by hematoxylin and eosin (H&E) staining. The expression levels of CD90 and HSP90 in the tumor were detected using immunohistochemical (IHC) staining. The inhibition rate (IR) of tumor volume was calculated as follows:

**TABLE 1:** Groups and treatments of targeted therapy of CD90@17-AAG/TMs.

**Groups**	**Treatments**
Control group	tumors injected with NS and unexposed to AMF
NS + AMF group	tumors injected with NS and exposed to AMF
TMs group	tumors injected with TMs and unexposed to AMF
TMs + AMF group	tumors injected with TMs and exposed to AMF
TSLs group	tumors injected with TSLs and unexposed to AMF
17-AAG/TSL group	tumors injected with 17-AAG/TSLs and unexposed to AMF
17-AAG/TMs +AMF group	tumors injected with 17-AAG/TMs and exposed to AMF
CD90@17-AAG/TMs +AMF group	tumors injected with CD90@17-AAG/TMs and exposed to AMF

IR of tumor volume = (1 − volume of experimental group/volume of control groups) × 100%.

IR of the tumor mass was calculated as follows:

IR of tumor mass = (1 − weight of experimental group/weight of control groups) × 100%.

The other half of the mice were fed until their natural death, and the survival time of each mouse in eight groups was recorded and plotted as a survival curve.

### Western Blotting Analysis

Tumor proteins were extracted from different groups and quantified at the end of the treatment. Protein extracts (40 μg) were separated by 12% sodium dodecyl sulfate polyacrylamide gel electrophoresis (SDS-PAGE) and transferred onto polyvinylidene fluoride membranes. Blots were blocked in 5% non-fat milk and incubated with anti-HSP90 (1:500, Zhong Shan Golden Bridge Biotechnology, Beijing, China) and β-actin (1:10,000, Sigma, St. Louis, MO, United States) antibodies at 4°C overnight. Blots were treated with a secondary antibody (1:10,000, Thermo, Waltham, MA, United States) for 1 h and visualized with SuperSignal^®^ West Pico Chemistry Luminescent Substrate (Thermo, Waltham, MA, United States).

### Statistical Analysis

Values represent the mean ± standard deviation (SD). The data were analyzed using the SPSS 16.0 software (IBM). A value of *p* < 0.05 was considered as statistically significant. All experiments were performed in triplicates.

## Results and Discussion

### Near-Infrared Imaging

Liposomes, as targeted drug delivery systems, have offered a new platform for therapies against CSCs. Liposomes modified with antisurface CSC antigens may not only ameliorate the *in vivo* drug distribution but also allow specific delivery of drugs to the target cells. Hence, establishing target evaluation systems is desirable.

**FIGURE 1:**
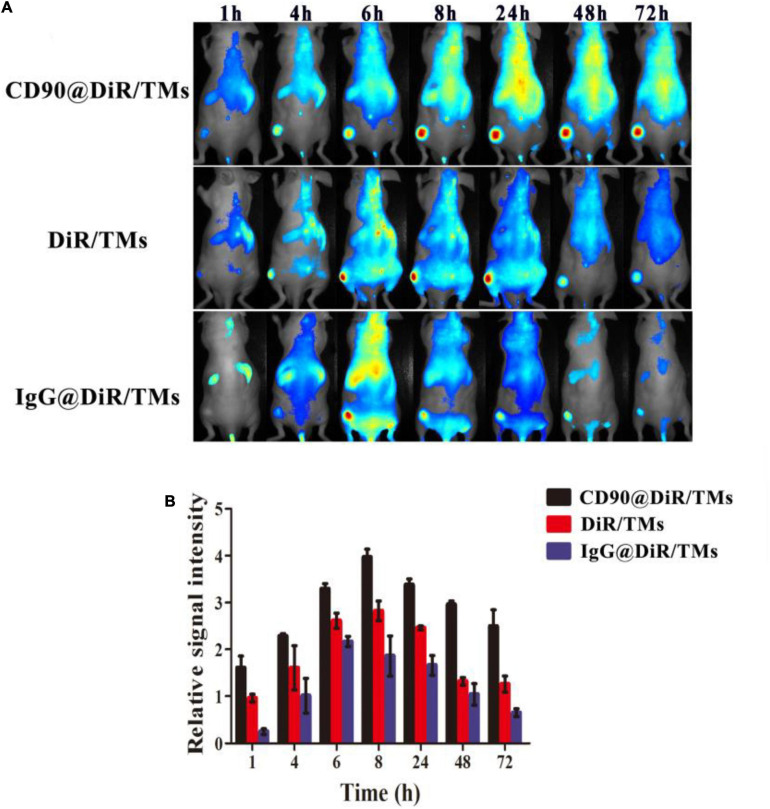
Targeting ability of CD90@DiR/TMs in CD90^+^ LCSC-bearing mice detected using NIR imaging. **(A)** NIR images at different time points following the injection of IgG@DiR/TMs, DiR/TMs, and CD90@DiR/TMs. **(B)** Relative fluorescence intensity of the tumor at different time points following the injection of IgG@DiR/TMs, DiR/TMs, and CD90@DiR/TMs.

The *in vivo* targeting ability of CD90@17-AAG/TMs was evaluated by the encapsulation of an NIR dye DiR into the liposomes (CD90@DiR/TMs). The accumulation of CD90@DiR/TMs, IgG@DiR/TMs, and DiR/TMs in the tumor tissue was observed using NIR imaging. The highest accumulation in the tumor was detected for the two groups from 6 to 8 h after tail vein injection with the same dose of DiR, and a gradual decrease in the signal intensity was observed after 8 h (Figure 1A). However, the fluorescence intensity reported for the tumor injected with CD90@DiR/TMs remained high for more than 1 h after injection (*p* < 0.05; Figure 1B). The fluorescence intensity reported for tumors from IgG@DiR/TM and DiR/TMs group significantly decreased over time. The relative fluorescence intensity observed for the tumor from CD90@DiR/TM group was higher than that observed for the tumor from the DiR/TM group at different time points (*p* < 0.05). The relative fluorescence intensity from 8 to 72 h was 1.5 times higher in the CD90@DiR/TM group than in the DiR/TM and IgG@DiR/TM groups, indicating that anti-CD90 mAb-modified liposomes may effectively bind to CD90^+^ LCSCs and show improved cellular uptake.

### Magnetic Resonance Imaging Analysis

Although the distribution of CD90@DiR/TMs in the tumor as well as in mice bodies can be studied using NIR fluorescent dyes, the spatial and temporal distribution may not be clearly observed. In addition, each drug has varying characteristics and specific location within cells, and NIR fluorescent dyes may be unable to simulate these features.

**FIGURE 2:**
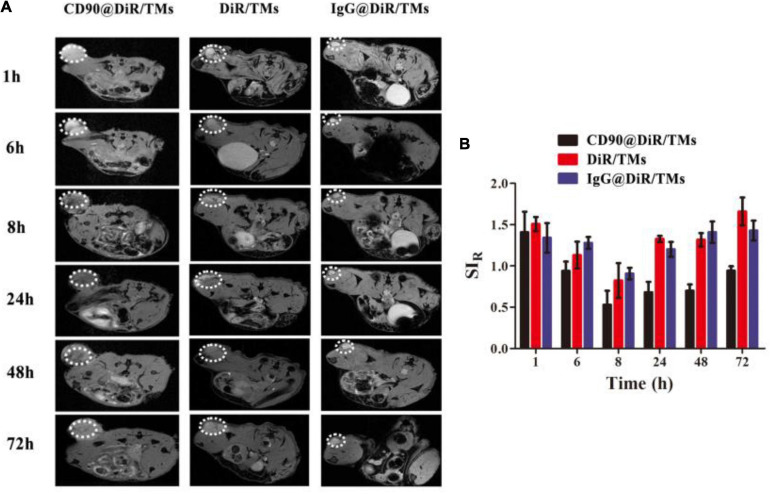
Targeting ability of CD90@DiR/TM in CD90^+^ LCSC-bearing mice observed using MRI. **(A)** MRI images at different time points following the injection of IgG@DiR/TMs, DiR/TMs, and CD90@DiR/TMs. **(B)** Relative signal intensities at different time points following the injection of IgG@DiR/TMs, DiR/TMs, and CD90@DiR/TMs.

Drugs used as imaging tracers should be more powerful and useful for the investigation of the interaction between drug-loaded liposomes and cells. To confirm the targeting ability of CD90@DiR/TMs, the cellular uptake of IgG@DiR/TMs, DiR/TMs, and CD90@DiR/TMs was studied using MRI based on the principle that ferric oxide (Fe_3_O_4_) effectively decreases the *T*_2_ relaxation time of the surrounding hydrogen proton (Hu et al., 2013). The accumulation of IgG@DiR/TMs, DiR/TMs, and CD90@DiR/TMs in the tumors increased and then gradually decreased over time, consistent with NIR imaging results (Figure 2A). The value of the relative signal intensity for tumors from the CD90@DiR/TM group was lower after 72 h of injection than that reported at 1 h after injection. In addition, the SI_*R*_ from 8 h was lower in the CD90@DiR/TM group than in the DiR/TMs and IgG@DiR/TMs (*p* < 0.05; Figure 2B). It means that the nanomaterials in the targeted group are more enriched in tumor tissues than in the non-targeted group and the irrelevant antibody group. In contrast, the relative signal intensities for tumors from the IgG@DiR/TM and DiR/TM groups showed no significant difference at the same time points (*p* > 0.05).

### Prussian Blue Staining

**FIGURE 3:**
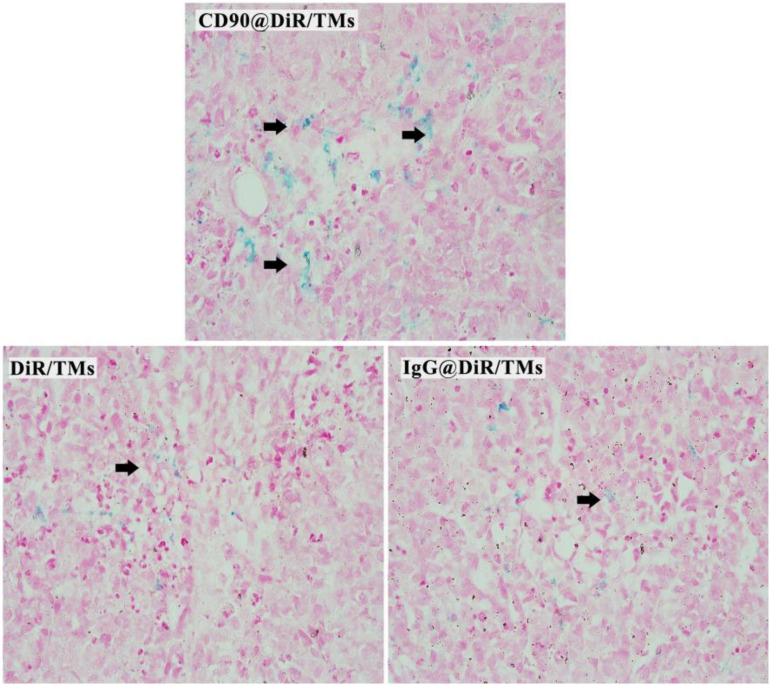
Prussian blue staining of the tumor tissues injected with DiR/TMs and CD90@DiR/TM (×40).

The targeting abilities of CD90@DiR/TMs, IgG@DiR/TMs, and DiR/TMs against CD90^+^ LCSCs in tumor tissues were further confirmed using Prussian blue staining, based on the principle that three-valent iron ions may react with potassium ferrocyanide to produce blue compounds under acidic conditions (Sluimer et al., 2015). The number of blue-stained particles in the tumor tissue from the CD90@DiR/TM group was significantly higher than that in the tumor tissue from the DiR/TM group (Figure 3). This result is consistent with NIR imaging and MRI results.

According to the enhanced permeability and retention (EPR) effect theory, the microvascular endothelial cells in human normal tissues are closely arranged; thus, polymeric drugs and lipid globules may not be able to penetrate through the blood vessels (Maeda et al., 2013). On the contrary, many solid tumors allow penetration of these particles as compared with normal tissues, thereby facilitating the extravasation of nanosized macromolecular anticancer drugs (particle size distribution range: 10–200 nm) owing to the abnormal vasculature in the rapidly proliferating tumors (Maeda, 2012). Polymeric drugs or lipid globules are easily intercepted due to the blockage of the lymphatic drainage. The particle size of the liposomes prepared was approximately 130 nm, which facilitated the easy accumulation of CD90@17-AAG/TMs in the tumor, attributable to the EPR effect and CD90 expression on the surface of LCSCs. Hence, Dir/TM and CD90@17-AAG/TMs were found to be distributed in the tumor using NIR imaging, MRI, and Prussian blue staining.

### *In vivo* Targeted Therapy With CD90@17-AAG/TMs

To enhance the treatment effect, tumors were treated with different therapies after 24 h of injection. NIR imaging and MRI results revealed CD90@17-AAG/TM enrichment and showed its ability to induce toxicity against non-LCSCs while affecting CD90^+^ LCSCs, owing to EPR effects and initial targeting function. The antitumor effect of CD90@17-AAG/TMs in CD90^+^ LCSC-bearing mice was first determined by calculating the tumor volume and tumor mass IRs. Table 2 shows that no significant differences were observed in tumor volume or tumor mass IRs among the normal saline (NS), NS + AMF, TM, and TSL groups; thus, AMF, TSLs, or TMs alone were non-toxic to tumors. The tumor volume and tumor mass IRs observed for the group treated with CD90@17-AAG/TMs in combination with hyperthermia were significantly higher than those reported for other groups (*p* < 0.05). The non-target group (17-AAG/TMs + AMF) also showed inhibition of tumor growth, possibly attributed to the enrichment of 17-AAG/TMs in the tumor tissues due to the EPR effect (Figures 1, 2). The combination of 17-AAG/TMs + AMF and hyperthermia may eliminate both LCSCs and non-LCSCs. Furthermore, the injection method (injection every other day) was conducive for better enrichment. The tumor volume and tumor mass IRs in the 17-AAG/TMs + AMF group were higher than those reported for the TMs + AMF and 17-AAG/TSL groups. This observation may be associated with the reversal of thermoresistance by 17-AAG, resulting in the apoptosis of tumor cells as previously reported (Yang et al., 2015).

**TABLE 2:** Tumor volume and tumor mass IRs in the different groups (%).

**No.**	**Groups**	**IRs of tumor volume ($\bar{x}$ ± s, *n* = 6)**	**IRs of tumor mass ($\bar{x}$ ± s, *n* = 6)**
1	NS	0	0
2	NS +AMF	0.47 ± 1.19^ac^	0.18 ± 1.16^ac^
3	TMs	1.08 ± 1.11^ac^	0.53 ± 1.62^ac^
4	TMs +AMF	24.53 ± 2.48^bc^	18.63 ± 2.42^bc^
5	TSLs	0.53 ± 1.12^ac^	0.19 ± 0.96^ac^
6	17-AAG/TSLs	21.27 ± 5.95^bc^	21.13 ± 5.02^bc^
7	17-AAG/TMs +AMF	64.13 ± 5.06^c^	41.7 ± 3.47^c^
8	CD90@17-AAG/TMs +AMF	88.0 ± 4.42^b^	76.53 ± 5.04^b^

*^a^Comparison of experimental groups with the TMs + AMF group, *p* < 0.05;*

*^*b*^comparison of experimental groups with the 17-AAG/TMs + AMF group, *p* < 0.05; ^*c*^comparison of experimental groups with the CD90@17-AAG/TMs +AMF group, *p* < 0.05.*

**FIGURE 4:**
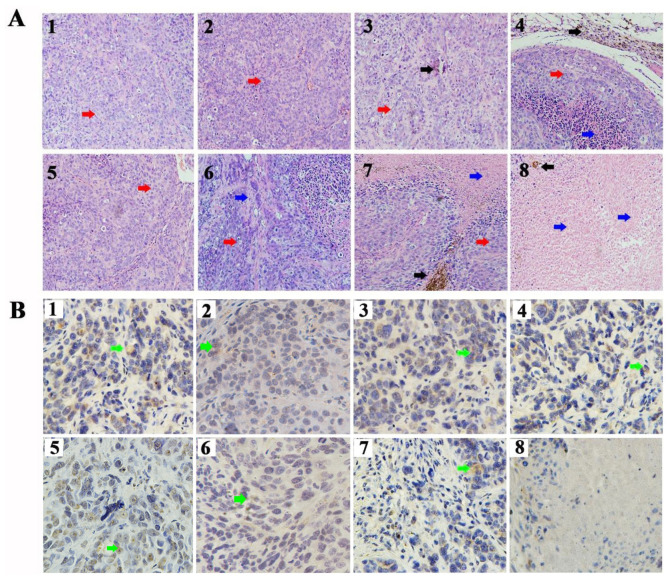
Targeted therapy of CD90@17-AAG/TMs in different groups. **(A)** H&E staining of the tumor tissues in different groups (×20, black arrows represent Fe_3_O_4_ nanoparticles, red arrows represent tumor tissues, and blue arrows represent necrotic tissue). **(B)** CD90 IHC staining of the tumor tissues in different groups (×40, green arrows represent the CD90-positive cells). 1. NS, 2. NS + AMF, 3. TMs, 4. TMs + AMF, 5. TSLs, 6. 17-AAG/TSLs, 7. 17-AAG/TMs + AMF, and 8. CD90@17-AAG/TMs + AMF.

To further confirm the therapeutic effect of CD90@17-AAG/TMs, H&E staining of the tumors was performed. Dark brown Fe_3_O_4_ sediment was observed in tumors treated with different TMs (Figure 4A). The non-targeted groups such as TMs + AMF, 17-AAG/TSLs, and 17-AAG/TMs + AMF caused varying degrees of damages to tumors, probably attributable to the EPR effect in tumor tissues. NS, NS + AMF, TM, and TSL groups showed no effect on tumor tissues. Necrosis was predominant in the CD90@17-AAG/TMs + AMF group, indicating that CD90@17-AAG/TMs in combination with hyperthermia may kill CD90^+^ LCSCs as well as CD90^–^ tumor cells. Tumor cells in this group were almost completely destroyed. CD90 expression was detected using IHC staining to confirm this hypothesis. CD90 expression in tumors treated with TMs + AMF, 17-AAG/TSLs, 17-AAG/TMs + AMF, and CD90@17-AAG/TMs decreased by varying levels (Table 3 and Figure 4B). The expression rate of CD90 in CD90@17-AAG/TMs was significantly lower than that in other groups (*p* < 0.05). Taken together, the results of H&E staining and CD90 IHC staining demonstrated the high killing ability of CD90@17-AAG/TMs against CD90^+^ LCSCs and CD90^–^ cells.

**TABLE 3:** CD90 expression rates in tumor tissue of mice (%).

**No.**	**Groups**	**Expression rates of CD90**
1	NS	31.8 ± 2.7
2	NS +AMF	34.9 ± 6.2^ac^
3	TMs	30.3 ± 3.0^ac^
4	TMs +AMF	17.9 ± 1.4^bc^
5	TSLs	34.2 ± 6.0^ac^
6	17-AAG/TSLs	16.3 ± 1.1^bc^
7	17-AAG/TMs +AMF	9.1 ± 1.2^c^
8	CD90@17-AAG/TMs +AMF	1.9 ± 2.5

*^*a*^Comparison of experimental groups with the NS group, *p* > 0.05; ^*b*^comparison of experimental groups with the 17-AAG/TMs + AMF group, *p* < 0.05; ^*c*^comparison of experimental groups with the CD90@17-AAG/TMs +AMF group, *p* < 0.05.*

**FIGURE 5:**
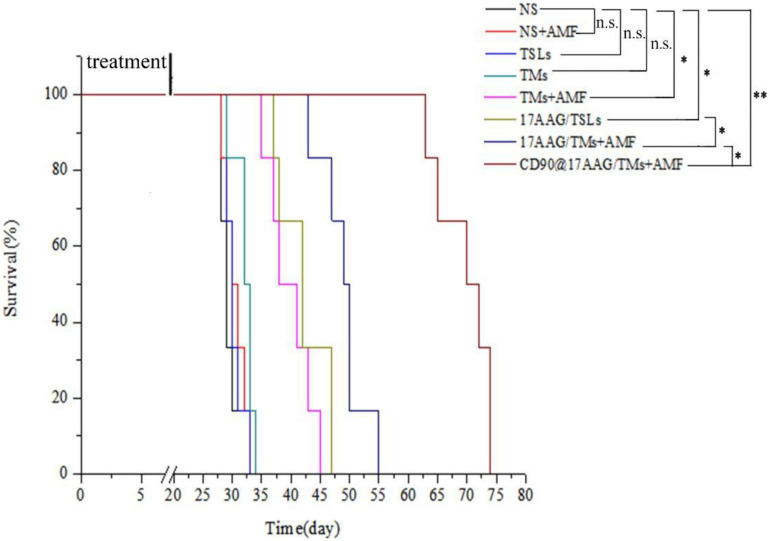
Kaplan–Meier survival curve of CD90^+^ LCSC-bearing mice in different groups. *n* = 6 mice per group. Data are the mean ± SD. ^∗^*p* < 0.05, ^∗∗^*p* < 0.01.

To evaluate the long-term effect of CD90@17-AAG/TMs + AMF therapy, overall survival of mice in each group was recorded. Kaplan–Meier survival curve was plotted according to the average survival time and SD (Figure 5). The results of the survival analysis were consistent with those of tumor volume IRs. No significant difference was observed between the NS, NS + AMF, TM, and TSL groups, suggestive of the absence of any effect of AMF, TSLs, or TMs on the survival time. The survival time was significantly longer for mice from the CD90@17-AAG/TM combined with hyperthermia group than those from other groups (*p* < 0.01). The non-targeted group (17-AAG/TMs + AMF) also showed prolonged survival as compared with the other four groups (*p* < 0.05) because of the inhibition ability reported due to the EPR effect. Furthermore, the survival time was longer for mice from the 17-AAG/TMs + AMF group than those from the TMs + AMF and 17-AAG/TSL groups (*p* < 0.05). All these results indicate that the tumor inhibition effect may transform into long-term survival benefit in CD90^+^ LCSC-bearing mice.

Given their involvement in tumor formation, progression, metastasis, recurrence, and therapeutic resistance, CSCs serve as an excellent target for cancer treatment. However, the tumor microenvironment is mostly composed of non-CSCs, which play an important role in protecting and regulating the growth of CSCs (Luo et al., 2015). Therapeutic strategies that target only CSCs or non-CSCs have several limitations. Comprehensive therapeutic agents affecting both CSCs and non-CSCs hold great potential to eliminate all tumor cells and completely cure cancer. CD90@17-AAG/TMs combined with hyperthermia effectively eliminated CD90^+^ LCSCs and CD90^–^ cells. Hence, we hypothesized that the therapeutic effect is attributed to the following reasons: CD90@17-AAG/TMs were enriched in the stroma of tumor cells and CD90^+^ LCSCs, owing to the EPR effect and CD90-targeting ability, which may be the most substantial reason for common elimination. The ability of tumor cells to engulf Fe_3_O_4_ is 4–800 times higher than that of normal cells. In addition, Fe_3_O_4_ endocytosed may be uniformly passed on to their progenies (Tay et al., 2018). If CD90@17-AAG/TMs were taken up by CD90^+^ LCSCs, the Fe_3_O_4_ particles may be transferred to their progenies (CD90^+^ LCSCs and CD90^–^ cells). AMF application may now kill both CD90^+^ LCSCs and CD90^–^ cells. The disintegration of CD90^+^ LCSC structure after treatment may result in the release of Fe_3_O_4_ particles into the surrounding environment, followed by their engulfment by other tumor cells (Jordan et al., 1999). Thus, multiple dosing and repeated treatments may potentially kill all types of tumor cells. The lysosomes from CD90^+^ LCSCs that have endocytosed CD90@17-AAG/TMs may show sudden and substantial destruction upon exposure to AMF (Baeza et al., 2013). This would result in the release and activation of lysosomal enzymes, which would damage CD90^+^ LCSCs and trigger injury to surrounding cells.

### Mechanism Underlying Antitumor Effects of CD90@17-AAG/TM in CD90^+^ LCSC-Bearing Mice

**FIGURE 6:**
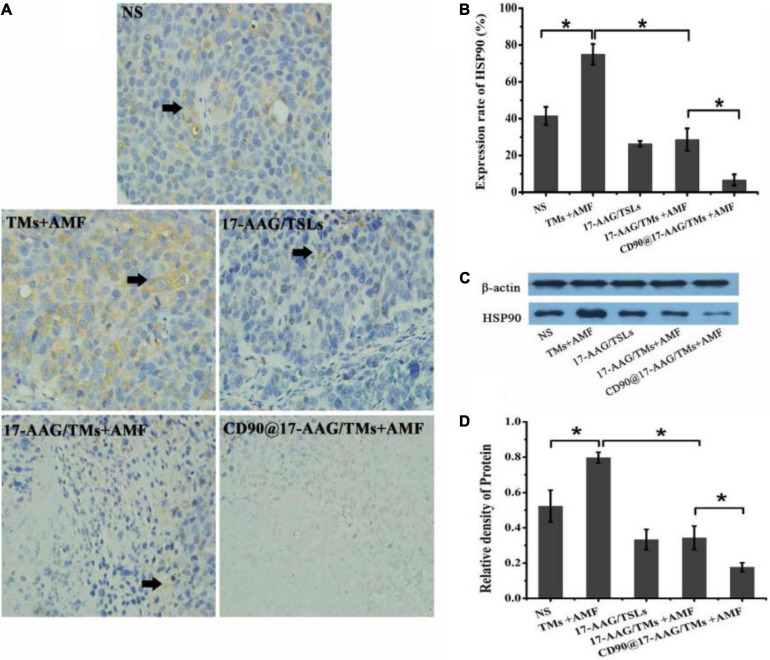
Antitumor mechanism of CD90@17-AAG/TMs in CD90^+^ LCSC-bearing mice. **(A)** HSP90 IHC staining of tumor tissues in different groups (×40). **(B)** Expression rate of HSP90, *p < 0.05. **(C)** HSP90 expression observed using Western blot analysis. **(D)** Relative density of HSP90, *p < 0.05.

Magnetic thermotherapy is a novel technique to heat deep-seated tumors and effectively kill CSCs. However, the application of hyperthermia alone has certain drawbacks, and the inclusion of heating time to enhance the effect of hyperthermia is more desirable. Thermotolerance may be developed in tumor cells undergoing multiheating treatment, resulting in reduced apoptosis and decreased cell death (Dai et al., 2013). HSPs are associated with thermoresistance and the induction of the heat shock response (Ahmed and Zaidi, 2013). The upregulation in HSP90 expression may affect the features or duration of thermotolerance (Millson and Piper, 2014). Inhibition of HSP90 may sensitize tumor cells to hyperthermia and result in increased apoptosis of tumor cells. 17-AAG is an HSP90 inhibitor derived from geldanamycin and may kill tumor cells by reversibly associating with HSP90 (Pacey et al., 2012). As a unique treatment, 17-AAG may effectively inhibit several cell signal transduction pathways involved in the maintenance of tumor cell proliferation and survival (Wang et al., 2014). To reduce the thermotolerance caused by magnetic thermotherapy, CD90@TMs encapsulating 17-AAG were prepared. The expression of HSP90 in NS, TMs + AMF, 17-AAG/TSLs, 17-AAG/TMs + AMF, and CD90@17-AAG/TMs was detected using HSP90 IHC staining and Western blot analysis. The expression rate of HSP90 was maximum in the TM + AMF group as compared to other groups (*p* < 0.05; Figures 6A,B), suggesting that multiheating treatment may induce thermotolerance. The encapsulation of 17-AAG in TMs resulted in a threefold decrease in the expression of HSP90 as compared to its encapsulation in TMs (28.7% ± 6.0% versus 75.0% ± 5.6%, *p* < 0.05), confirming the association between 17-AAG and HSP90^+^ cells to overcome thermotolerance and induce cell death. The group treated with CD90@17-AAG/TMs showed minimum HSP90 expression, suggestive of the application of HSP90 to sensitize tumor cells to hyperthermia to achieve increased cell death. HSP90 protein quantitative test was performed using Western blot analysis. The trend observed in the relative expression of HSP90 coincided with the results of IHC staining. The relative intensity of HSP90 in the TM group was significantly higher than that observed in the NS and 17-AAG/TM groups (*p* < 0.05; Figures 5C,D), indicating that hyperthermia may upregulate the expression of HSP90 and that 17-AAG could effectively sensitize HSP90^+^ tumor cells to magnetic hyperthermia. This observation also explains why the combination of 17-AAG/TMs and hyperthermia was more effective than the combination of TM and hyperthermia.

Hepatocellular carcinoma is one of the most common malignant tumors in the world, especially in Southeast Asia (Johnson et al., 2013). Although surgery and radiofrequency ablation were used to treat HCC, and the common idea of these treatments is to decrease the volume of tumor tissues and the number of tumor cells in the greatest extent, recurrence is common after a certain period of time (Peng et al., 2012; Wald et al., 2013; Kwon et al., 2015). Hepatic artery embolism chemotherapy is the main treatment for advanced liver cancer and has a remission rate of only 30% (Song et al., 2015). Liu et al. (2018) synthesized the ^131^I-labeled copper sulfide-loaded microspheres and combined embolization therapy, chemotherapy, radiotherapy, and photothermal therapy to treat hepatic tumors via hepatic artery embolization; the results showed that the embolization therapy in combination with chemotherapy, radiotherapy, and photothermal therapy could completely ablate the transplanted hepatic tumors *in situ*, while embolization combined with one or two therapy modalities slowed but did not stop tumor growth (Liu et al., 2018). In our study, the hyperthermia combined with one therapy modality can also inhibit the growth of tumor. One of the differences in our study is that the tumor model is ectopic. In future study, we will use orthotopic HCC model to verify the efficacy of CD90@17-AAG/TMs. In addition, the common occurrence of drug resistance often leads to chemotherapy failure. The first-line and second-line treatments for HCC, including sorafenib, regorafenib, and lenvatinib, can effectively treat HCC when combined with radiation therapy and chemotherapy. However, a study showed that the therapeutic effects are limited because of the high recurrence and drug resistance of LCSCs (Vu et al., 2013; Liu et al., 2020). The CSC theory explains the reason for treatment failure and demands new targets and orientation for HCC therapy. LCSCs cause tumor formation, recurrence, metastasis, and drug resistance (Chen et al., 2013). Hence, the permanent cure necessitates the complete eradication of CSCs. However, therapeutic strategies for CSCs are not different from those for non-CSCs. Non-CSCs have a protective effect on the growth of CSCs. In the present study, we enhanced the therapeutic effect against CD90^+^ LCSCs by subjecting tumors to CD90@17-AAG/TM therapy every other day to strengthen the EPR effect and increase the endocytosis of liposomes. The treatment was continued for 7 days. CD90@17-AAG/TMs in combination with thermotherapy effectively killed CD90^+^ LCSCs and CD90^–^ cells *in vivo*. Although this study focused on the application of CD90@17-AAG/TMs in the treatment of HCC, other anticancer drugs could be encapsulated in these nanoparticles to eliminate CSCs and non-CSCs.

## Conclusion

In this study, we constructed an anti-CD90 mAb-modified 17-AAG-loaded magnetic thermosensitive liposome (CD90@17-AAG/TMs) to target killing liver cancer cells. *In vivo*, it can remain in the tumor tissue through the CD90. The treatment results showed that CD90@17-AAG/TMs can effectively kill CD90^+^ LCSCs and CD90^–^ cells and inhibit tumor growth. In summary, CD90@17-AAG/TMs have great potential and application value in the targeted treatment of liver cancer.

## Data Availability Statement

The raw data supporting the conclusions of this article will be made available by the authors, without undue reservation.

## Ethics Statement

The animal study was reviewed and approved by Institutional Animal Care and Use Committee (IACUC) of Southeast University.

## Author Contributions

QT was contributed to the design and conception of the subject. YA gave great support in animal imaging. XW is responsible for the implementation of the experiments, the results sorting, and manuscript writing. RY and YH gave lots of guidances on experiments details and manuscript submission. CH, ZZ, and DL was helpful in flow cytometry, animal model construction, and pictures processing. All authors contributed to the article and approved the submitted version.

## Conflict of Interest

The authors declare that the research was conducted in the absence of any commercial or financial relationships that could be construed as a potential conflict of interest. The reviewer WW declared a shared affiliation, with no collaboration, with one of the author ZZ to the handling editor at the time of the review.

## Publisher’s Note

All claims expressed in this article are solely those of the authors and do not necessarily represent those of their affiliated organizations, or those of the publisher, the editors and the reviewers. Any product that may be evaluated in this article, or claim that may be made by its manufacturer, is not guaranteed or endorsed by the publisher.
