# Corrigendum: Facile assembly of thermosensitive liposomes for active targeting imaging and synergetic chemo-/magnetic hyperthermia therapy

**DOI:** 10.3389/fbioe.2025.1579340

**Published:** 2025-05-06

**Authors:** Yanli An, Rui Yang, Xihui Wang, Yong Han, Gang Jia, Chunmei Hu, Zhiyuan Zhang, Dongfang Liu, Qiusha Tang

**Affiliations:** ^1^ Jiangsu Key Laboratory of Molecular and Functional Imaging, Department of Radiology, Zhongda Hospital, Medical School of Southeast University, Nanjing, China; ^2^ Research Institute for Reproductive Health and Genetic Diseases, The Affiliated Wuxi Maternity and Child Health Care Hospital of Nanjing Medical University, Wuxi, China; ^3^ School of Medicine, Southeast University, Nanjing, China; ^4^ Department of Tuberculosis, The Second Affiliated Hospital of Southeast University (The Second Hospital of Nanjing), Nanjing, China; ^5^ Department of Neurosurgery, Nanjing Jinling Hospital, School of Medicine, Nanjing University, Nanjing, China

**Keywords:** CD90, combined therapy, imaging, LCSCs, hyperthermia therapy

In the published article, there was an error in Figure 4 as published. The IHC staining images of Figure 4B for “5” does not correspond to the correct groups. The corrected Figure 4 and its caption FIGURE 4 Targeted therapy of CD90@17-AAG/TMs in different groups. (A) H&E staining of the tumor tissues in different groups (×20, black arrows represent Fe_3_O_4_ nanoparticles, red arrows represent tumor tissues, and blue arrows represent necrotic tissue). (B) CD90 IHC staining of the tumor tissues in different groups (×40, green arrows represent the CD90-positive cells). 1. NS, 2. NS + AMF, 3. TMs, 4. TMs + AMF, 5. TSLs, 6. 17-AAG/TSLs, 7. 17-AAG/TMs + AMF, and 8.CD90@17-AAG/TMs + AMF. appear below.

**FIGURE 4:**
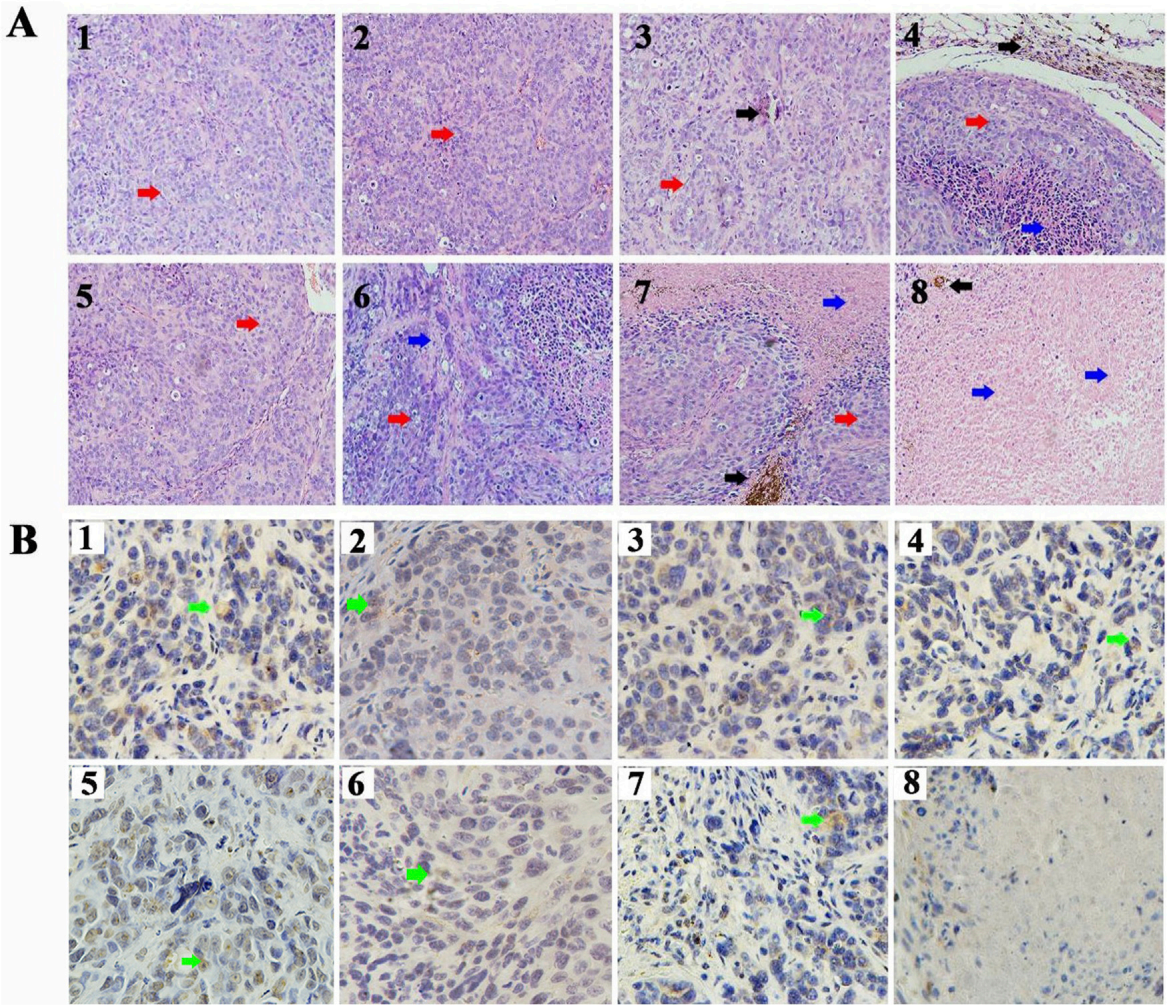
Targeted therapy of CD90@17-AAG/TMs in different groups. **(A)** H&E staining of the tumor tissues in different groups (×20, black arrows represent Fe_3_O_4_ nanoparticles, red arrows represent tumor tissues, and blue arrows represent necrotic tissue). **(B)** CD90 IHC staining of the tumor tissues in different groups (×40, green arrows represent the CD90-positive cells). 1. NS, 2. NS + AMF, 3. TMs, 4. TMs + AMF, 5. TSLs, 6. 17-AAG/TSLs, 7. 17-AAG/TMs + AMF, and 8.CD90@17-AAG/TMs + AMF.

The authors apologize for this error and state that this does not change the scientific conclusions of the article in any way. The original article has been updated.

